# An Emerging Perspective on the Role of Fascia in Complex Regional Pain Syndrome: A Narrative Review

**DOI:** 10.3390/ijms26062826

**Published:** 2025-03-20

**Authors:** Carmelo Pirri, Nina Pirri, Lucia Petrelli, Caterina Fede, Raffaele De Caro, Carla Stecco

**Affiliations:** 1 Department of Neuroscience, Institute of Human Anatomy, University of Padova, 35121 Padova, Italy; lucia.petrelli@unipd.it (L.P.); caterina.fede@unipd.it (C.F.); rdecaro@unipd.it (R.D.C.); carla.stecco@unipd.it (C.S.); 2 Department of Medicine—DIMED, School of Radiology, Radiology Institute, University of Padua, 35121 Padova, Italy; nina_92_@hotmail.it

**Keywords:** superficial fascia, deep fascia, blood vessels, nervous autonomic system, free nerve endings, pain, oxidative stress

## Abstract

Complex Regional Pain Syndrome (CRPS) is a debilitating pain disorder involving chronic inflammation, neural sensitization and autonomic dysfunction. Fascia, a highly innervated connective tissue, is increasingly recognized for its role in pain modulation, yet its contribution to CRPS remains underexplored. This narrative review synthesizes the current evidence on fascia’s involvement in CRPS pathophysiology and potential therapeutic strategies. A literature search was conducted in PubMed, Scopus and Web of Science, selecting studies on fascia, CRPS, inflammation, oxidative stress and autonomic dysfunction, with emphasis on recent experimental, anatomical and clinical research. Fascia contributes to CRPS through neuroinflammation, fibrosis and autonomic dysregulation. Its rich innervation facilitates peripheral and central sensitization, while inflammatory mediators drive fibrosis, reducing elasticity and exacerbating pain. Autonomic dysfunction worsens hypoxia and oxidative stress, fueling chronic dysfunction. Advances in sonoelastography provide new insights, while fascial manipulation and targeted therapies show promise in early studies. Fascia plays a key role in CRPS pathophysiology, yet its clinical relevance remains underexplored. Future research integrating imaging, molecular profiling and clinical trials is needed to develop evidence-based fascia-targeted interventions, potentially improving CRPS diagnosis and treatment.

## 1. Introduction

Fascia is a ubiquitous connective tissue that envelops muscles, nerves, organs and blood vessels, providing both structural support and functional integration throughout the body [1]. It is composed primarily of collagen fibers, which endow it with strength and flexibility, allowing it to adapt to movements and stresses [2]. Fascia plays a key role in maintaining musculoskeletal stability and transmitting mechanical forces, but its functions extend beyond mere physical support. It is richly innervated and contains numerous mechanoreceptors and free nerve endings, making it a significant player in proprioception and pain sensation [3,4,5,6,7]. Recent research has highlighted fascia’s involvement in various chronic pain conditions, where fascial dysfunction may contribute to pain sensitization, restricted mobility and inflammation [8,9,10,11]. Fascia is a dynamic and highly specialized tissue, characterized by a layered structure that includes both superficial and deep fasciae [12]. The superficial fascia is situated just beneath the skin, providing a protective and energy-storing layer that divides the subcutaneous tissue into superficial and deep adipose tissue compartments, while the deep fascia envelops muscles, tendons and bones, playing a crucial role in force transmission and movement coordination [1]. The deep fascia can be further divided into aponeurotic and epimysial layers. Aponeurotic fasciae are large sheets that envelop multiple muscle groups, acting as key structures in the continuity of force transmission, whereas epimysial fasciae specifically cover individual muscles and are tightly bound to their surface [2]. Recent findings have revealed that fascia is not simply a passive structure; rather, it has a dense network of neural elements, including nociceptors, proprioceptors and autonomic fibers, which play an important role in the modulation of pain and proprioceptive functions [13].

Fascia also contains a variety of cellular components, including fibroblasts, which are responsible for the maintenance and remodeling of the extracellular matrix [14]. These fascial fibroblasts respond to biochemical and mechanical stimuli, which allows the fascia to adapt to changes in mechanical load. Recent studies have demonstrated that fascia is involved in the inflammatory process and may play a role in the progression of fibrosis through the activity of the renin–angiotensin system, which regulates extracellular matrix remodeling [15]. This adaptability is crucial in maintaining the functional integrity of muscles and joints, particularly under conditions of mechanical stress or injury, emphasizing fascia’s role in musculoskeletal health beyond simple structural support.

Complex Regional Pain Syndrome (CRPS) is a multifaced and debilitating chronic pain condition, often triggered by trauma or surgery, that is characterized by disproportionate pain in an affected region. Despite decades of research, CRPS remains a challenging pain disorder, and its underlying mechanisms remain elusive, with contributions from autoimmune, neuroinflammatory and psychosocial factors [16]. While extensive research has explored its neuropathic and neuroimmune components, emerging evidence suggests that fascial alterations may play a significant yet under-recognized role in CRPS pathophysiology. However, this relationship remains poorly defined, with conflicting findings in the literature. Fascia has recently garnered attention in the study of chronic pain syndromes, like CRPS, integrating current research into its contributions to both symptom development and potential treatment avenues. Unlike previous reviews that have primarily focused on the neurogenic and inflammatory mechanisms of CRPS [16,17], this review uniquely integrates anatomical, molecular and clinical perspectives on the role of fascia in CRPS. By synthesizing recent experimental and clinical findings, this study aims to provide a more comprehensive understanding of the interplay between fascia, peripheral sensitization and chronic pain. This perspective is critical for advancing research and guiding the development of more targeted therapeutic approaches, including US-guided fascial interventions and novel rehabilitative techniques. Addressing the gap in the evidence linking fascial dysfunction to CRPS pathophysiology is essential to refine both diagnostic and therapeutic strategies.

## 2. Materials and Methods

A comprehensive literature review was conducted to explore the current evidence on Complex Regional Pain Syndrome (CRPS) and its relationship with fascia, particularly focusing on the roles of superficial fascia and deep fascia in pain mechanisms. An extensive search strategy was developed and applied across PubMed, Scopus and Web of Science databases, using the following keywords and Boolean combinations: “(Complex Regional Pain Syndrome AND superficial fascia AND pain mechanisms) OR (Complex Regional Pain Syndrome AND deep fascia AND inflammation) OR (Complex Regional Pain Syndrome AND fascia AND chronic pain) OR (Complex Regional Pain Syndrome AND fascial anatomy AND pain management) OR (Complex Regional Pain Syndrome AND superficial fascia AND pain sensitivity) OR (Complex Regional Pain Syndrome AND myofascial dysfunction AND deep fascia) OR (Complex Regional Pain Syndrome AND fascia AND neuroinflammation) OR (Complex Regional Pain Syndrome AND fascial layers AND rehabilitation) OR (Complex Regional Pain Syndrome AND fascia AND pain modulation)”.

**Figure 1:**
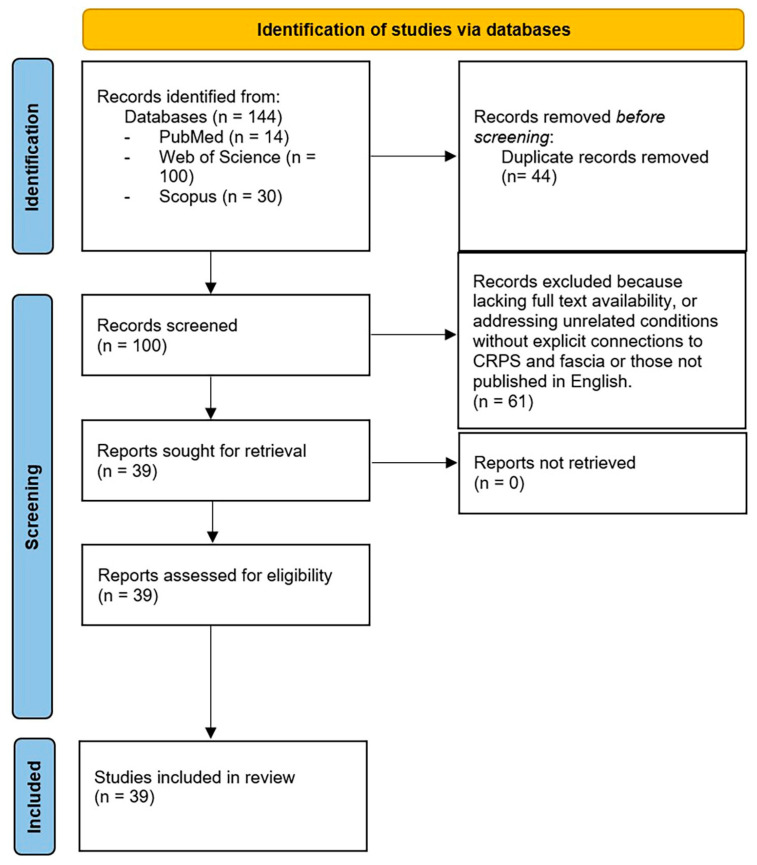
Flowchart of study selection.

The inclusion criteria encompassed all types of peer-reviewed articles, ensuring a broad capture of studies related to the interplay between CRPS and fasciae. Two independent reviewers (C.P. and N.P.) screened the titles and abstracts of the identified studies, with a third reviewer (C.S.) resolving disagreements and finalizing the selection process. The initial search retrieved 144 publications. After excluding non-English studies and irrelevant articles, the dataset was curated, where duplicate entries were systematically removed. This process resulted in 100 articles for further evaluation. Each title and abstract were independently assessed by the reviewers, culminating in the inclusion of 39 publications for detailed analysis (Figure 1).

The selected studies highlighted key findings regarding the role of fascia in CRPS pathophysiology, particularly the involvement of superficial and deep fasciae. The evidence suggests that fasciae play a critical role in neuroinflammatory processes, pain transmission and the maintenance of myofascial dysfunction on CRPS. Emerging insights indicate that alteration in the fasciae contributes to pain sensitization, reduced mobility and functional impairments observed in CRPS patients. Although disagreements during the review process were resolved by consensus, no formal quality assessment or bias evaluation was conducted for the included studies because it is a narrative review. Nevertheless, this review provides an updated synthesis of the intricate relationship between CRPS and fasciae, emphasizing their significance in both understanding pain mechanisms and guiding future therapeutic strategies.

## 3. Current Knowledge About Pathophysiology of CRPS

The pathophysiology of CRPS is multifactorial, involving a combination of peripheral and central mechanisms, as well as contributions from the autonomic and immune systems. CRPS begins with an initial inciting event, such as an injury, fracture or surgery, which initiates a cascade of pathological responses. These responses include inflammatory reactions, altered pain processing and autonomic dysregulation, which together contribute to the complexity of CRPS symptoms. One of the earliest processes in CRPS is excessive local inflammation. The initial trauma can lead to a robust inflammatory response characterized by the release of pro-inflammatory cytokines, such as IL-1, IL-6 and TNF-α. These cytokines promote vascular permeability, resulting in swelling and redness, and sensitize peripheral nociceptors, which leads to hyperalgesia (an increased response to painful stimuli) and allodynia (pain from normally non-painful stimuli). Other inflammatory mediators, including bradykinin, substance P and prostaglandins, are also released, further intensifying the sensation of pain [17,18,19]. This inflammatory milieu not only perpetuates the pain but also sets the stage for subsequent tissue damage and fibrosis.

The autonomic nervous system plays a crucial role in the pathophysiology of CRPS. Following the initial trauma, there is often an aberrant increase in sympathetic nervous system activity. This heightened activity can lead to vasomotor dysfunction, manifesting as abnormal temperature regulation, skin discoloration and sweating in the affected limb. Some authors have suggested that CRPS may follow distinct phases, where the affected area initially appears warm and hyperemic, while in later stages, reduced sympathetic tone may lead to cold, cyanotic skin [20,21,22,23,24]. However, other studies propose that CRPS may not progress through clear stages but rather presents as distinct “warm” and “cold” subtypes, each with different pathophysiological mechanisms [25]. Dysregulated sympathetic activity can also cause persistent vasoconstriction, leading to reduced blood flow, tissue hypoxia and the development of fibrosis in the fascia and surrounding tissue [26]. These structural changes contribute to a vicious cycle of pain, autonomic dysfunction and tissue remodeling, making CRPS a condition that is difficult to treat and interrupt.

Central sensitization is another critical factor contributing to the chronicity and intensity of CRPS. Central sensitization refers to an increased responsiveness of the neurons in the central nervous system (CNS), particularly within the spinal cord and brain, to both noxious and non-noxious stimuli. This phenomenon results in an exaggerated pain response and can cause pain to spread beyond the original site of injury [26,27]. Neuroplastic changes, such as cortical reorganization in the somatosensory cortex, have been observed in CRPS patients, indicating that prolonged pain can lead to significant alterations in brain structure and function, further exacerbating the experience of pain and contributing to the spread of symptoms [28].

The immune system also plays a pivotal role in the pathophysiology of CRPS. There is growing evidence suggesting that CRPS may have an autoimmune component. Immune cells, such as macrophages, T cells and mast cells, infiltrate the affected tissues, releasing cytokines that promote neuroinflammation [29]. Furthermore, autoantibodies against specific components of the nervous system have been identified in a subset of CRPS patients, suggesting that an autoimmune response may be contributing to the persistence of inflammation and neuronal damage [30]. This immune involvement adds another layer of complexity to CRPS, making it not just a disorder of the nervous system but also a condition with significant immune dysregulation.

Oxidative stress has also been implicated in the pathophysiology of CRPS. The combination of reduced blood flow, increased metabolic activity of immune cells and ongoing inflammation creates an environment of oxidative stress in the affected tissues. Reactive oxygen species (ROS) can cause further tissue damage and amplify pain signaling pathways, contributing to the chronic nature of CRPS [31,32,33]. This oxidative damage can lead to mitochondrial dysfunction within neurons and muscle cells, exacerbating energy deficits and promoting further neuronal injury [34,35,36,37,38]. Taken together, the pathophysiology of CRPS is characterized by a complex interplay between inflammation, autonomic dysregulation, central sensitization, immune responses and oxidative stress [31,32,33,34,35,36,37,38]. This multifactorial nature explains the variability in symptoms and the difficulty in developing a standardized treatment protocol that addresses all aspects of the syndrome. Understanding these underlying mechanisms is crucial for developing targeted therapies that not only alleviate symptoms but also address the root causes of CRPS. Approaches that modulate inflammation, reduce central sensitization, restore autonomic balance and mitigate immune dysfunction hold promise for more effective management of this challenging condition (Table 1).

## 4. Innervation of Fasciae

**Figure 2:**
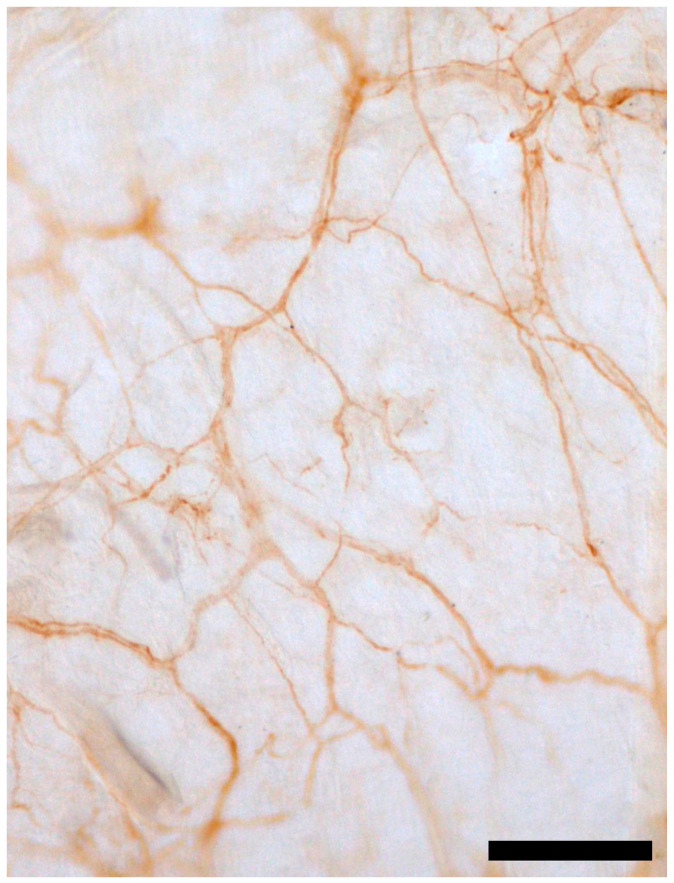
Innervation of mouse thoracolumbar floating samples stained with S100 antibody. Scale bars: 200 μm.

The role of fasciae in pain and autonomic dysfunction has emerged as a critical focus in understanding the pathophysiology of conditions like CRPS. Beyond its traditional view as a passive structural tissue, fascia is increasingly recognized for its active contribution to sensory and autonomic processes. This recognition stems from its dynamic properties, which allow it to influence inflammation, pain modulation and neuromuscular interactions. Fasciae are connective tissues that envelop muscles, organs and other structures, traditionally thought to provide only mechanical support. The recent evidence, however, has transformed this perspective, demonstrating that fasciae are richly innervated tissues with significant sensory and autonomic roles [13,39,40]. These findings underline their involvement in pain perception, proprioception and even thermoregulation, prompting a re-evaluation of their physiological and pathological significance [13,39,40]. The current understanding of fascial innervation has been significantly enhanced through immunohistochemical studies, which reveal intricate networks of nerve fibers and their distribution across different fascia types. These advancements have implications for treating chronic pain, particularly myofascial pain syndromes, and guiding surgical and manual therapies (Figure 2).

The superficial fascia is situated within the subcutaneous tissue, composed of fibro-fatty connective tissue interspersed with adipocytes and blood vessels. Recent histological analyses reveal a dense network of both thin and thick nerve fibers within this layer with a notable concentration near blood vessels and adipose tissue. Immunohistochemical staining using S100 and PGP 9.5 antibodies demonstrates significant sensory and autonomic innervation, with autonomic fibers constituting approximately 33% of the total innervation [28]. These findings suggest a role for the superficial fascia in thermoregulation and pain perception, potentially mediated through interactions with the autonomic nervous system. The presence of large nerve bundles (mean diameter 21.1 ± 12.2 µm) and smaller fibers (mean diameter 4.8 ± 2.6 µm) supports its classification as a highly innervated tissue with direct involvement in sensory functions [31]. Superficial fascia also demonstrates functional plasticity through its relationship with surrounding structures. The innervation observed includes fibers reactive to tyrosine hydroxylase, indicative of autonomic activity, and sensory fibers that penetrate fibroadipose tissue and connect with blood vessels. This dual innervation underscores its potential in mediating systemic physiological responses, including stress-related modulation of vascular tone and sensitivity to external mechanical stimuli [13]. Deep fasciae, subdivided into aponeurotic and epimysial types, display distinct patterns of innervation. The thoracolumbar (TLF), an aponeurotic fascia, exhibits a dense network of free nerve endings, contributing to its role in proprioception and pain modulation [13]. Quantitative studies reveal that the TLF has a higher density of innervation (9.01% of the analyzed area) compared to the gluteal fascia (2.78%), an epimysial fascia. Free nerve endings in the TLF are particularly sensitive to chemical and mechanical stimuli, underlying their involvement in non-specific low back pain [38]. Unlike the TLF, the gluteal fascia shows a more limited role in pain modulation but plays a significant part in coordinating muscle activity through its connections with Golgi tendon organs and muscle spindles [34]. For instance, muscle spindles within the perimysium further enhance this coordination, highlighting the epimysial fascia’s role in integrating motor unit actions [41].

The autonomic innervation of the TLF and gluteal fascia quantified at 0.08% for both, suggests a shared role in vasomotor regulation. However, the marked differences in free nerve ending density indicate divergent sensory roles, with the TLF acting as a proprioceptive hub responsive to mechanical stimuli from the associated muscle groups [13,38,39,40,41]. Moreover, the absence of mechanoreceptors, like Pacini and Ruffini corpuscles, in these fasciae, as confirmed in multiple studies, implies that their sensory input is predominantly mediated through free nerve endings sensitive to stretch and shear forces [13,38,39,40,41] (Table 2).

## 5. Vascularization of Fasciae

In addition to their sensory and mechanical roles, fasciae are deeply involved in vascular and neurovascular dynamics, which are increasingly recognized as critical in the pathophysiology of CRPS. Altered vascularization within fasciae may exacerbate key pathological processes in CRPS, including inflammation, hypoxia and oxidative stress, thereby intensifying pain and dysfunction. Understanding the vascular architecture of fasciae is essential, as it underpins their roles in thermoregulation, nutrient delivery and neurovascular communication.

**Figure 3:**
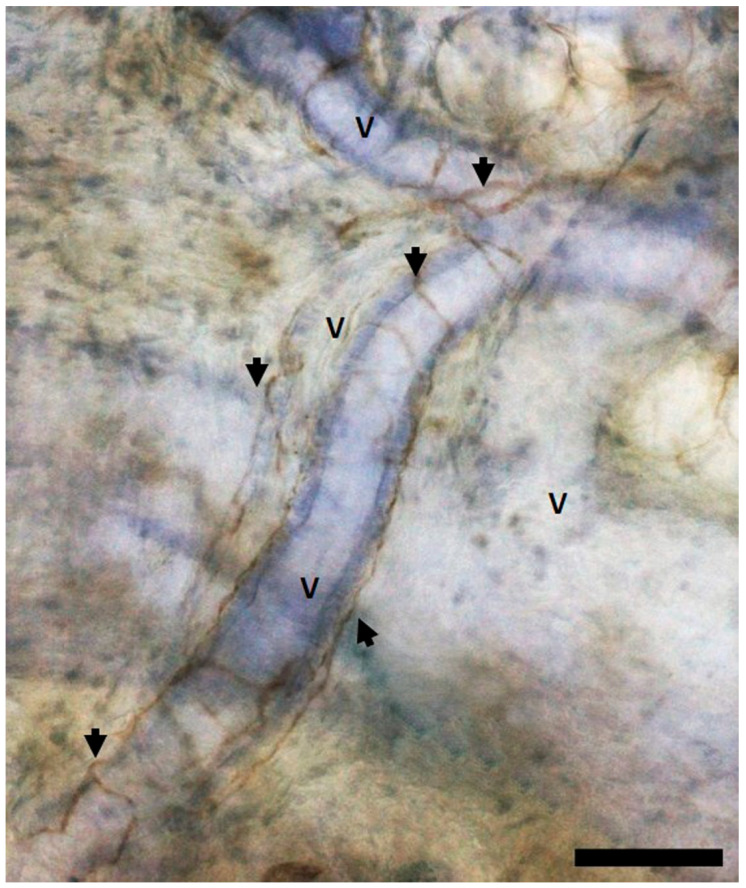
Human superficial fascia vasculature in free-floating sections. Samples stained with S100 and hematoxylin. v: vessels; arrows: innervation. Scale bars: 100 μm.

The vascularization of fasciae, In particular, superficial fascia, represents a key determinant of their structural and physiological roles. Recent studies have illuminated the intricate vascular networks within fasciae, underscoring their importance in thermoregulation, nutrient delivery and neurovascular interactions. Scarpa’s fascia, a prominent example of superficial fascia located in the anterior abdominal wall, has been shown to host a dense vascular meshwork comprising arteries, veins, capillaries and lymphatic vessels. Quantitative immunohistochemical analyses reveal that approximately 6.20% of Scarpa’s fascia is occupied by von Willebrand factor-positive vessels, with an additional 2.93% accounted for by smooth muscle actin-positive vessels, indicative of arteriolar components [42]. The vascular architecture of fasciae is characterized by a highly organized and homogeneously distributed network (Figure 3).

The vessels traverse the connective tissue longitudinally and transversely, forming a rete mirabilis that optimizes hemodynamic flow and metabolic exchange. Vessel diameters range from 13 to 65 µm, with arteries averaging 54.24 ± 15.80 µm and veins at 60.10 ± 1.04 µm, while capillaries and lymphatic vessels are smaller than 20 µm in diameter [42]. Fractal analysis has further confirmed this distribution, demonstrating an optimal spatial organization with a fractal dimension of 1.063 ± 0.10 and lacunarity of 0.60 ± 0.10. These metrics highlight the efficiency of the vascular network in occupying the fascia’s space, ensuring functional integration with the surrounding tissue [41,42]. One notable aspect of fascia vascularization is the presence of arterio-venous anastomoses (AVAs), which directly link small arteries to veins without intervening in the capillary bed. These anastomoses not only enhance circulatory efficiency but also play a critical role in thermoregulation, allowing rapid adjustments to temperature fluctuations. This role is supported by the autonomic innervation surrounding these vessels, which regulates the vasomotor tone through the release of mediators, such as nitric oxide and endothelin, facilitating precise control of vasodilatation and vasoconstriction [42,43]. The vascularization of fasciae is deeply integrated with the autonomic nervous system, highlighting the dynamic interplay between vascular and neural components. Vascular innervation within superficial fascia consists of tyrosine hydroxylase-positive and S100-positive fibers, indicative of the sympathetic modulation of vasomotor responses. These neurovascular interactions are crucial for maintaining systemic homeostasis, enabling rapid responses to mechanical stimuli and regulating blood flow [42,43]. Additionally, the presence of renin-angiotensin system components within fasciae, including angiotensin II receptors, underscores the role of fasciae in broader cardiovascular regulation [42,43]. The vascularization of fasciae exemplifies the dynamic nature of these tissues, transcending their traditional role as structural scaffolds. The complexity and integration of the vascular network, in particular, its neurovascular interplay, emphasize fascia’s role in maintaining homeostasis and responding to environmental and physiological demands (Table 3).

## 6. Role of Fascia in CRPS

**Figure 4:**
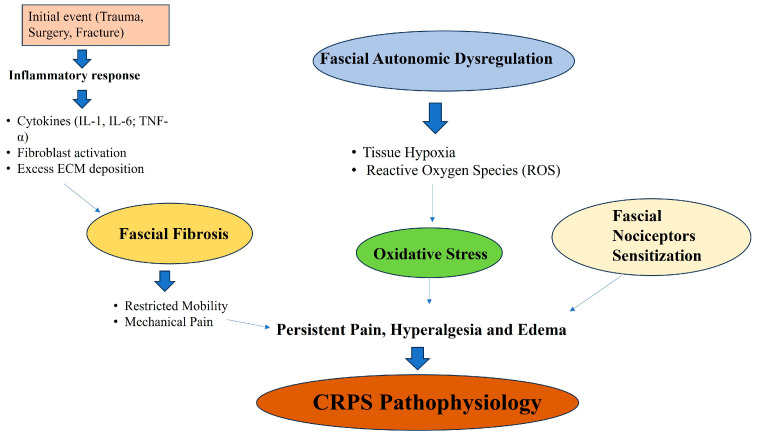
Schematic description of fascial role in the pathophysiology of Complex Regional Pain Syndrome.

The intricate role of fascia in the pathophysiology of CRPS has gained increasing attention due to its dynamic nature and ability to influence pain, inflammation and autonomic functions. Fascia is no longer viewed as a passive structural tissue but rather an active participant in neuromuscular health, capable of modulating sensory and autonomic responses. Fascia is highly vascularized and innervated, making it susceptible to inflammatory mediators released during trauma or persistent pain states, such as CRPS. After an inciting trauma, such as fracture, surgery or soft tissue injury, a cascade of pro-inflammatory mediators, including cytokines, such as IL-1, IL-6 and TNF-α, is triggered, stimulating fibroblast and autonomic activation. This leads to reparative processes, such as the deposition of a new extracellular matrix and collagen fibers, neoangiogenesis and cell proliferation. In reality, in terms of a physiological condition, this reaction stops in a few days, allowing a reduction in inflammation and the remodeling of the tissue. In some cases, the perpetuation of the fibroblasts and autonomic activation leads to excessive extracellular matrix deposition and fibrosis. Fascial fibrosis contributes to restricted mobility, stiffness and mechanical pain, which are hallmarks of CRPS. Recent studies also indicate the renin-angiotensin system within fascia plays a critical role in tissue remodeling [15]. These cytokines increase vascular permeability, causing swelling and pain while simultaneously activating peripheral nociceptors embedded in the fascia. The inflammatory response is further amplified by immune cells within the fascia, such as macrophages and mast cells, which release additional mediators, like histamine and prostaglandins. These substances perpetuate nociceptive sensitization and create an environment conducive to chronic inflammation [32,44,45]. Simultaneously, fascial fibroblasts are stimulated by inflammatory mediators to produce an excess extracellular matrix (ECM), in particular, collagen. While collagen deposition is critical for tissue repair, excessive ECM remodeling leads to fibrosis, reducing the fascia’s elasticity and impairing its function in force transmission and movement [13,38,39,40,41]. Fascial fibrosis not only restricts mobility but also creates mechanical stress on the surrounding tissues, contributing to persistent mechanical pain, which is a hallmark symptom of CRPS. This ongoing inflammatory and fibrotic process forms a vicious cycle where fascial dysfunction exacerbates pain and mobility issues, further fueling the pathology of CRPS (Figure 4).

Fascia is densely innervated by nociceptors, making it a critical structure for pain modulation. In CRPS, inflammatory mediators, such as substance P, bradykinin and prostaglandins, sensitize nociceptors within the fascia, reducing their activation threshold. This process of peripheral sensitization leads to hyperalgesia and allodynia. These sensitized nociceptors provide a continuous stream of nociceptive input to the spinal cord and brain, driving central sensitization [46]. Studies have demonstrated that mechanical stress or tension in the TLF can generate substantial nociceptive input, which is then amplified by central sensitization mechanisms. Neuroplastic changes in the CNS, such as cortical reorganization in the somatosensory cortex, have been observed in CRPS patients, further amplifying pain perception and enabling the spread of pain to regions beyond the original site of injury [47,48]. Fascia is richly innervated by sympathetic fibers, connecting it to autonomic nervous system and making it a key player in CRPS-associated autonomic dysfunction. After trauma, abnormal sympathetic activity leads to vasomotor instability, causing irregular blood flow, impaired thermoregulation and tissue hypoxia within the fascia. Some authors have suggested that CRPS may follow a progression of stages, where a hyperactive sympathetic tone initially manifests as warmth, redness and hyperemia in the affected region. However, others propose that CRPS may not necessarily evolve through well-defined stages but instead exist as two distinct subtypes: a “warm” type, characterized by vasodilation and hyperemia, and a “cold” type, marked by vasoconstriction and ischemia [26]. Over time, sympathetic dysfunction can result in persistent vasoconstriction, leading to cold, cyanotic skin, chronic ischemia and impaired tissue perfusion [49]. This autonomic dysregulation affects fascia, as reduced blood flow triggers tissue hypoxia and the generation of reactive oxygen species (ROS). These ROS not only damage cellular components but also activate nociceptive pathways, perpetuating pain, inflammation and fascial dysfunction.

In addition, the hypoxic environment stimulates fibroblast activity, further driving fibrosis and limiting fascial elasticity. Autonomic dysfunction within fascia also contributes to the abnormal sweating patterns and temperature dysregulation commonly observed in CRPS patients [9,50]. The superficial fascia is characterized by a higher density of autonomic fibers and vascular structures compared to the deep fascia, making it more susceptible to mechanisms involving autonomic and vascular influences, whereas the deep fascia is predominantly affected by fibrotic processes, contributing to tissue stiffness. Fascial fibrosis is a hallmark of chronic CRPS, driven by the persistent activation of fibroblasts and excess ECM deposition. This fibrotic transformation reduces the fascia’s compliance, impairing its ability to stretch and adapt to mechanical forces during movement. The loss of fascial elasticity creates mechanical stress on underlying muscles, tendons and joints, exacerbating pain and restricting mobility. These mechanical changes are not limited to the affected region; fascial adhesions and stiffness can alter force transmission across connected structures, contributing to widespread musculoskeletal dysfunction [51].

The interplay between fascial fibrosis and neural sensitization creates a feedback loop where restricted movement and mechanical tension in the fascia amplify nociceptive signaling, which, in turn, perpetuates fibrosis. This explains why CRPS patients often report worsening pain with movement or physical therapy. Targeting fascial fibrosis through interventions such as manual therapy and pharmacological treatments could break this cycle and restore functional mobility [51].

Oxidative stress is a key contributor to CRPS pathophysiology, with fascia serving as a site of both ROS generation and damage, and hypoxia within the fascia, driven by impaired blood flow and chronic inflammation, creates an environment conducive to the production of ROS [52]. These ROS cause oxidative damage to cellular components, including mitochondria, leading to energy deficits and impaired tissue repair. Mitochondrial dysfunction within fascial fibroblasts and nearby muscle cells could further exacerbates pain and tissue degradation [53,54]. Oxidative stress also sensitizes nociceptive pathways within the fascia, amplifying pain signaling and contributing to the chronic nature of CRPS (Table 4).

While emerging evidence supports the possible involvement of fascial alterations in CRPS pathophysiology, it is crucial to acknowledge studies that propose alternative mechanisms. Central sensitization has been identified as a significant factor, characterized by widespread pain hypersensitivity and hyperexcitable central neurons. This suggests that central nervous system dysfunction may play a pivotal role in CRPS, potentially independent of peripheral and fascial changes [55]. Moreover, neuroinflammation has been implicated as a primary contributor to CRPS pain, with localized inflammatory processes in the nervous system potentially sustaining the condition without direct fascial involvement [56]. These different viewpoints highlight the complexity of CRPS and the necessity for comprehensive research to clarify the relative contributions of fascial alterations and peripheral sensitization, central sensitization and neuroinflammatory processes in its pathogenesis.

**Table 4:** Papers about role of fasciae in CRPS. N/A = Not Applicable.

Study	Type of Study	Randomization	Subjects (M/F)	Age Range	Focus	Treatments	Readouts for Evaluation	Key Messages	Notes
Sinhorim et al. (2021) [44]	Scope review	N/A	N/A	Various	Thoracolumbar fascia	Various	Nociceptive role assessment	Fascia may contribute to pain processing	Review of in vivo/ex vivo studies
Zullo et al. (2021) [45]	Molecular study	N/A	N/A	Various	Fibrosis and myofibroblast differentiation	Various	Role of sirtuins in fibrosis	Sirtuins as checkpoints in fibrosis modulation	Target for therapeutic intervention
Schmidt et al. (2024) [46]	Field study	N/A	Human subjects	Various	Nociplastic pain	Clinical application	Pain grading system validation	Applicability of nociplastic pain grading	Pain classification improvement
Ortiz et al. (2024) [47]	Experimental study	N/A	Mice model	Various	Peripheral inflammation	Fascia manipulation	Adenosine A1 receptor mediation	Fascia manipulation induces analgesia	Mechanistic insights
Chapman et al. (2021) [48]	Experimental study	Yes	Human subjects	Various	Dorsal root ganglion stimulation	Neuromodulation	Lead migration prevention	New approach to reduce complications	Clinical relevance
Forero et al. (2022) [49]	Case study	N/A	Human subject	Adult	CRPS type I	Erector spinae plane block	Long-term pain relief	Sustained analgesia for 2 years	Single case report
Hoheisel et al. (2015) [9]	Experimental study	N/A	Rats	N/A	Fascia inflammation	Induced inflammation	Innervation alterations	Changes in fascia innervation patterns	Preclinical study
Weinkauf et al. (2015) [50]	Experimental study	N/A	Human and animal models	Various	NGF-induced sensitization	Neuromodulation	Sensitization analysis	Differences in muscle vs. fascia responses	Preclinical relevance
Zügel et al. (2018) [51]	Consensus statement	N/A	N/A	Various	Sports medicine and fascia	Tissue adaptation	Injury and diagnostics framework	Fascial research recommendations	Multidisciplinary consensus
Pirri et al. (2022) [52]	Clinical case report	N/A	Human subjects	Various	Rigid retinacula in CRPS	Ultrasound and fascial manipulation	Imaging and treatment response	Improved pain and function	Fascial therapy insights
Taha & Blaise (2012) [53]	Review	N/A	N/A	Various	CRPS pathogenesis	Various	Role of oxidative stress	Identified oxidative stress mechanisms	Foundational review
Guo et al. (2018) [54]	Animal study	N/A	Rodents	Various	CRPS model	Fracture/cast-induced inflammation	Pain and inflammation assessment	Oxidative stress contributes to CRPS	Animal model insights
Schranz et al. (2020) [57]	Case report	No	1 (1 M)	Not specified	Manual therapy for CRPS	Counterstrain therapy	Symptom resolution, proprioception improvement	Counterstrain therapy improved proprioception and reduced symptoms	Single case report; needs further validation
Marrone et al. (2024) [58]	Review	N/A	Multiple studies	Various	Fascial plane blocks for chronic pain	Fascial plane blocks	Pain relief, effectiveness in chronic pain management, including CRPS	Growing evidence supporting fascial plane blocks, but more studies needed	Calls for larger clinical trials
Benkli et al. (2023) [59]	Case report	No	1 (1 M)	Not specified	Refractory post-surgical neuralgia and CRPS	ESP catheter under US + fluoroscopy	Pain scores, opioid consumption	ESP catheterization reduced pain by 80% and opioid use by 50%	Long-term follow-up showed lasting benefits
Bang et al. (2023) [60]	Case report	No	2 (1 M/1 F)	Not specified	ESP catheterization in CRPS	ESP catheterization	Pain relief duration, opioid reduction	ESP catheterization effective for 14 days, reducing opioid use by 50%	Limited to case reports; further trials needed

Although large-scale clinical trials validating fascia-targeted therapies for CRPS are still lacking, some case reports suggest potential clinical benefits [49,52,57,58,59,60]. For example, in two CRPS cases, B-mode Ultrasound (US) Imaging and sonoelastography identified stiff retinacula linked to symptoms, and both patients improved significantly following Fascial Manipulation^®^ therapy [52]. Similarly, a case report demonstrated that counterstrain therapy resulted in sustained symptom resolution in a CRPS patient, along with improvements in proprioception and thermal discrimination [57]. Moreover, a recent review highlighted the role of US-guided fascial plane blocks in chronic pain, including CRPS [57]. Benkli et al. described a case of refractory post-surgical neuralgia after aneurysm rupture and craniotomy, treated with an erector spinae plane (ESP) block [59]. Forero et al. reported the treatment of CRPS type 1 with ESP catheter implantation under combined US and fluoroscopic guidance. A 2-year follow-up demonstrated an 80% reduction in pain scores from baseline and a 50% reduction in opioid consumption [49]. Similarly, Bang et al. [60] reported two CRPS cases, in which patients experienced significant pain relief for 14 days following ESP catheterization, with opioid consumption reduced by 50%. These findings suggest that interventional approaches targeting the fascial planes may offer valuable pain relief strategies for CRPS. Despite these promising observations, the evidence remains limited, emphasizing the need for randomized controlled trials (RCTs) to establish standardized treatment protocols and assess long-term efficacy.

### Future Perspectives in Diagnostics and Therapy of CRPS

The understanding of fascia’s role in CRPS offers exciting opportunities for advancing diagnostics, therapies and research into this complex condition. One key area is the development of advanced imaging technologies, such as ultrasound elastography and diffusion tensor imaging, to detect fascial changes, including fibrosis, stiffness or inflammation. These tools could enable non-invasive assessments of fascial health, improving diagnostic precision and facilitating early detection of CRPS-related changes. Furthermore, molecular-level investigations into the cellular behavior of fibroblasts, immune cells and neural elements within fascia may uncover new therapeutic targets. For example, understanding how fibroblasts drive fibrosis or how nociceptors and mechanoreceptors contribute to pain amplification could pave the way for interventions that directly modulate these processes. Exploring inflammatory and oxidative stress pathways in fascia could also yield biomarkers for disease progression and treatment response, allowing for more personalized approaches to care. Therapeutically, innovations such as targeted pharmacological treatments to reduce fibrosis and antioxidants to mitigate oxidative damage and regenerative therapies to restore fascial function represent promising directions. Furthermore, integrating fascial health into rehabilitation strategies—through physical and manual therapies aimed at restoring mobility and reducing stiffness—has the potential to transform patient outcomes. Collaborative, multidisciplinary research that bridges molecular biology, imaging science and clinical practice will be essential for translating these insights into effective solutions for CRPS. By prioritizing fascia as a therapeutic target, future advancements can address the root causes of pain and dysfunction, offering hope for more effective and lasting relief for patients.

## 7. Conclusions

Fascia could play an emerging central role in the pathophysiology of CRPS, influencing and being influenced by inflammation, neural sensitization, autonomic dysfunction, fibrosis and oxidative stress. Its dynamic interplay with the processes highlights fascia as both a mediator and a target in CRPS. Understanding the intricate mechanisms by which fascia could contribute to CRPS not only deepens our comprehension of this complex condition but also provides a foundation for innovative treatment strategies. Therapies targeting fascial dysfunction—ranging from manual interventions and pharmacological treatments to exercise programs and antioxidant therapies—have the potential to alleviate symptoms, disrupt pathological feedback loops and restore function in CRPS patients. Future research should focus on elucidating the precise molecular pathways involved and evaluating the long-term efficacy of fascial-targeted interventions, paving the way for more effective and comprehensive management approaches.

## Figures and Tables

**Table 1:** Pathophysiological mechanisms of CRPS.

Mechanism	Description	Implications	Key References
**Inflammatory Response**	CRPS is initiated by an excessive local inflammatory reaction following trauma. Pro-inflammatory cytokines, such as IL-1, IL-6 and TNF-α, contribute to increased vascular permeability, leading to swelling, redness, hyperalgesia and allodynia. Other mediators, like bradykinin, substance P and prostaglandins, further intensify pain.	Persistent inflammation perpetuates pain and contributes to long-term tissue damage and fibrosis. Targeting inflammatory pathways may provide therapeutic benefits.	[17,18,19]
**Autonomic Nervous System Dysregulation**	Following trauma, the sympathetic nervous system exhibits abnormal activity, resulting in vasomotor dysfunction. Some authors suggest that CRPS may progress through stages, with early manifestations of increased sympathetic tone, leading to hyperemia and warmth, while later phases may be characterized by reduced sympathetic activity, resulting in cold, cyanotic skin. However, others propose that CRPS consists of distinct “warm” and “cold” subtypes rather than a linear progression. Regardless of classification, persistent vasoconstriction contributes to tissue hypoxia and fibrosis, perpetuating pain and dysfunction.	Autonomic dysfunction plays a critical role in the persistence of CRPS. Understanding whether the condition progresses through stages or exists as subtypes is crucial for tailoring treatments. Interventions targeting sympathetic regulation, including pharmacologic, interventional and rehabilitative approaches, may help alleviate symptoms.	[20,21,22,23,24,25,26]
**Central Sensitization**	Enhanced excitability of neurons in the spinal cord and brain leads to exaggerated pain responses, even from non-noxious stimuli. Neuroplastic changes, including cortical reorganization, result in pain spreading beyond the original injury site.	Central mechanisms contribute significantly to CRPS chronicity. Addressing central sensitization with NMDA receptor antagonists and neuromodulation may be beneficial.	[27,28]
**Immune System Involvement**	Macrophages, T cells, and mast cells infiltrate affected tissues, releasing pro-inflammatory cytokines that promote neuroinflammation. Autoantibodies against neural structures suggest an autoimmune contribution to CRPS.	Immunomodulatory therapies targeting cytokine release and autoantibody production are potential avenues for treatment.	[29,30]
**Oxidative Stress**	Reduced blood flow, increased immune activity and ongoing inflammation create a state of oxidative stress. Reactive oxygen species (ROS) amplify pain signaling and contribute to tissue damage. Mitochondrial dysfunction exacerbates energy deficits, leading to neuronal injury.	Antioxidant therapies and mitochondrial-targeted treatments may reduce oxidative damage and improve symptoms.	[31,32,33,34,35,36,37,38]
**Multifactorial Nature of CRPS**	CRPS arises from the interplay between inflammation, autonomic dysfunction, central sensitization, immune dysregulation and oxidative stress. This complexity explains symptom variability and treatment resistance.	Comprehensive, multi-targeted treatment approaches are necessary to effectively manage CRPS. Personalized therapeutic strategies may yield the best outcomes.	[31,32,33,34,35,36,37,38]

**Table 2:** Papers about innervation of fasciae. N/A: Not Applicable.

Study	Type of Study	Randomization	Subjects (M/F)	Age Range	Onset of CRPS	Treatments	Readouts for Evaluation	Key Messages	Notes
Fede et al. (2022) [38]	Anatomical study	N/A	Human samples	N/A	N/A	Histological examination	Fascia innervation	Identified neural pathways in fascia	Fundamental anatomy study
Suarez-Rodriguez et al. (2022) [13]	Systematic review	N/A	N/A	Various	Various	Various	Fascia innervation review	Reviewed literature on fascial innervation	Literature review
Fede et al. (2021) [39]	Experimental study	N/A	Mice samples	N/A	N/A	Neural network analysis	Evidence of hidden neural networks	Found new neural networks in fascia	Foundational study
Fan et al. (2021) [40]	Experimental study	N/A	Mice samples	Various	Various	ECM analysis	Hyaluronan and collagen alterations	Showed ECM changes with aging	Aging study
Stecco et al. (2023) [41]	Review	N/A	N/A	Various	Various	Various	Myofascial unit concept	Proposed integrated view of myofascial units	Future research direction

**Table 3:** Papers about vascularization of fasciae. N/A: Not Applicable.

Study	Type of Study	Randomization	Subjects (M/F)	Age Range	Focus	Treatments	Readouts for Evaluation	Key Messages	Notes
Stecco et al. (2023) [41]	Review	N/A	N/A	Various	Myofascial unit	Various	Structural and functional insights	Defines myofascial unit	Future research
Pirri et al. (2023) [42]	Anatomical study	N/A	Human Samples	N/A	Fascia blood supply	Dissection-based study	Analysis of vascular patterns	Provides anatomical basis for fascia perfusion	Foundational study
Yamashiro & Yanagisawa (2020) [43]	Molecular study	N/A	N/A	Various	Mechanotransduction	Various	Molecular signaling	Explores vascular homeostasis	Molecular mechanisms discussed
